# Somatostatin Receptor Scintigraphy in Autoimmune Syndrome Induced by Silicone Breast Implants: Pre- and Postexplantation Findings

**DOI:** 10.3390/jcm14124141

**Published:** 2025-06-11

**Authors:** Luz Kelly Anzola, Sara Ramirez, Sergio Moreno, Camilo Vargas, Sebastian Rojas, José Nelson Rivera

**Affiliations:** 1 Nuclear Medicine Unit, Clinica Reina Sofia, Bogotá 110121, Colombia; 2 Nuclear Medicine Unit, Clinica Universitaria Colombia, Bogotá 110931, Colombia; 3 Fundación Universitaria Sanitas, Bogotá 110131, Colombia; saritaramirezaguirre@gmail.com (S.R.); s.rojasla@unisanitas.edu.co (S.R.); 4 Clinical Research Institute, Universidad Nacional de Colombia, Bogotá 111321, Colombia; smmorenol@unal.edu.co; 5 Medicina Nuclear Sanitas Palermo, Bogotá 111311, Colombia; cc.vargaslo@unisanitas.edu.co; 6 Internal Medicine Department, Clinica Reina Sofia, Bogotá 110121, Colombia; jonerimo@hotmail.com

**Keywords:** ASIA syndrome, silicone breast implants, ^99m^Tc-HYNIC-TOC scintigraphy, autoimmune inflammation, explantation, somatostatin receptor imaging

## Abstract

**Background:** Silicone breast implants have been linked to autoimmune/inflammatory syndrome induced by adjuvants (ASIA). This study evaluates the role of ^99m^Tc-HYNIC-TOC somatostatin receptor scintigraphy in assessing somatostatin-mediated inflammation and the impact of explantation on inflammatory activity. **Methods:** Fifty patients with silicone breast implants and symptoms suggestive of ASIA were evaluated. Pre- and postexplantation imaging was performed using ^99m^Tc-HYNIC-TOC scintigraphy. Matthews correlation coefficients quantified associations between clinical symptoms and imaging findings, and autoantibody profiles were analysed. **Results:** Scintigraphy identified a significant uptake in organs associated with autoimmune symptoms, particularly joints and salivary glands. Strong correlations were found between imaging findings and symptoms, including knee pain (MCC = 0.81) and sicca syndrome (MCC = 0.96). Explantation resolved abnormal uptake in the surgical bed, though variable uptake persisted in other organs, reflecting systemic inflammatory heterogeneity. Autoantibody analysis revealed positivity in 66% of patients, with antinuclear antibodies being most frequent (30%). **Conclusions:** ^99m^Tc-HYNIC-TOC scintigraphy effectively evaluates organ-specific inflammation in ASIA. Explantation reduces localized inflammation but does not consistently address systemic autoimmune responses. Larger prospective studies are needed to validate these findings and improve management strategies for ASIA.

## 1. Introduction

The term “autoimmune/inflammatory syndrome induced by adjuvants” (ASIA), which emphasizes the role of environmental factors in the development of immune-mediated diseases, was introduced by Shoenfeld in 2011 [1]. He identified four conditions sharing similar signs and symptoms, all linked to prior exposure to an adjuvant: silicone-induced disease, Gulf War syndrome, macrophagic myofasciitis syndrome, and postvaccination phenomena. In genetically susceptible individuals, exposure to environmental factors such as infections, toxins, and drugs can trigger not only the onset of immune-mediated conditions but also influence their clinical manifestations [2]. Environmental agents with immune adjuvant effects, including silicone, aluminium, vaccines, and infections, have long been recognized. These adjuvants have been shown to induce autoimmunity in various animal models and may trigger autoinflammatory diseases in humans [3]. An immunologic adjuvant is defined as a substance that enhances antigen-specific immune responses without eliciting a response on its own [4]. The adjuvant effect operates through mechanisms affecting both innate and adaptive immunity; enhancing the activities of dendritic cells, lymphocytes, and macrophages; increasing local reactions to antigens; and promoting the release of chemokines and cytokines from T-helper and mast cells [5].

Over the past two decades, increasing evidence has linked the presence of silicone implants to subsequent autoimmune-related complications [6]. Studies suggest that while some patients develop postexposure autoimmune phenomena such as ASIA, others remain unaffected [7]. The identified at-risk groups for developing ASIA after receiving silicone implants include individuals with prior autoimmune reactions to adjuvants (e.g., vaccines, implants), those with established autoimmune conditions (e.g., Hashimoto’s disease, Graves’ disease, rheumatoid arthritis), individuals with a history of allergic or atopic disorders (e.g., eczema, pollen allergy, drug allergy), and those genetically predisposed to autoimmunity (e.g., HLA-DR4, HLA-DRB1) [8]. Silicone breast implants (SBIs) were initially believed to be durable; however, it was later discovered that the implant sheath can degrade over time, allowing silicone to migrate into surrounding tissues [9]. Yu et al. estimated that silicone diffuses at approximately 300 mg per year [10]. The adverse effects in patients with SBIs are extensive and include local complications such as lymphadenopathy, skin lesions, and pericapsular chronic inflammation [11]. Additionally, the direct impact of silicone on cellular immunity may lead to delayed-type hypersensitivity reactions involving Th1/Th17 cells, resulting in systemic symptoms associated with inflammatory and autoimmune diseases. T cells surrounding the silicone capsule often produce interleukin-17, interleukin-6, interleukin-8, and other growth factors [12]. The adjuvant effects on the humoral immune system are evidenced by increased levels of anti-collagen autoantibodies [13], elevated antinuclear antibodies (ANAs), and increased IL-2 levels in patients with SBIs, along with findings from animal models showing elevated anti-DNA antibodies [14]. Interest in the health effects of SBIs has resurfaced in the past decade because of reports of BI-associated anaplastic large-cell lymphoma (BIA-ALCL), desmoid tumours dependent on a periprosthetic capsule, and associations with autoimmune diseases [15]. Recent guidelines emphasize the importance of assessing antithyroid antibodies, rheumatoid factor, the erythrocyte sedimentation rate (ESR), immunoglobulin levels, and C-reactive protein as part of the preoperative evaluation to identify patients predisposed to developing autoimmune disorders or those with subclinical autoimmune diseases [16]. The detection of ANAs associated with Sjögren’s syndrome, the most common autoimmune condition linked to SBIs, is also recommended [17]. While the role of molecular imaging in this context has not been extensively evaluated, its potential in detecting inflammation related to autoimmune conditions is noteworthy. Advances in imaging technologies have introduced promising tools for diagnosing and monitoring these clinical conditions. Examples include macrophage CD206 receptors radiolabelled with 99mTc-Tilmanocept for diagnosing active rheumatoid arthritis [18], interleukin-2 radiolabelled with 99mTc-HMPAO for identifying active Crohn’s disease [19], and 2-deoxy-18F-fluoro-9-β-D-arabinofuranosylguanine for imaging T-cell activation [20]. Additionally, researchers have explored the use of radiolabelled somatostatin (SST) receptor 2 (SSTR 2) for detecting Sjögren’s syndrome and rheumatoid arthritis [21,22,23], among other conditions. The high diagnostic accuracy of these analogues stems from the strong binding affinity of SSTRs for their five receptor subtypes, which are expressed in various cells, including lymphocytes, monocytes, and inflammatory cells [24,25]. SST acts as a regulatory hormone that influences several physiological processes and plays a significant role in inflammatory responses. Clinical applications of radiolabelled SST analogues have yielded significant results in evaluating disease activity, prognosis, and therapeutic response in chronic inflammatory diseases [26,27]. Molecular imaging targeting SSTRs is a promising diagnostic tool for identifying active inflammation across various disorders and provides unique in vivo histopathological insights into cell-mediated inflammatory processes [22]. Recent studies have confirmed the ability of SSTR scintigraphy to localize active inflammation in both symptomatic and asymptomatic joints, as well as extra-articular sites such as salivary glands, demonstrating its potential in assessing conditions such as Graves’ ophthalmopathy, Sjögren’s disease, and rheumatoid arthritis [21,28,29,30]. Understanding how SBIs can provoke autoimmune responses is essential, as the immune reaction to silicone, although generally benign, may exacerbate underlying conditions in susceptible individuals. This underscores the necessity for personalized patient counselling and thorough risk assessment.

The primary objective of this study was to describe the pattern of uptake of SSTR scintigraphy in a cohort of patients with autoimmune symptoms and SBIs. The secondary objective was to describe findings from pre- and postexplantation studies in a small cohort of patients. We hypothesized that 99mTcHYNIC-TOC receptor scintigraphy can be used to identify organs with SST-mediated inflammation in patients with SBIs presenting with autoimmune symptoms.

## 2. Materials and Methods

### 2.1. Study Design

This study involved a descriptive analysis of 50 individuals with suspected ASIA associated with SBIs. An analytical cross-sectional study was conducted with retrospective data to describe the pattern uptake of radiolabelled SST ^99m^TcHYNIC-TOC scintigraphy. The study recruited participants from the nuclear medicine department from January 2019 to November 2023. A subset of seven patients underwent postexplantation scintigraphy, allowing for a comparison of pre- and postexplantation imaging findings. This research was approved by the hospital’s Ethics Committee (approval number: 2293-23 CEIFUS). Owing to the retrospective nature of the analysis, the requirement for written informed consent from the patients was waived.

### 2.2. Study Population

A nonprobabilistic consecutive sampling method was used; patients with SBIs and ASIA symptoms were referred by the clinician to the nuclear unit of the institution to assess inflammatory activity by using radiolabelled SSTR scintigraphy. All patients presented with anamnestic or clinical presentations of possible autoimmune conditions, and serological markers of immunological disease were obtained. All patients underwent ^99m^T-HYNIC-TOC scintigraphy.

### 2.3. Radiopharmaceutical

^99m^TcHYNIC-TOC was prepared in the radiopharmaceutical department from a commercially available kit (Tektrotyd^®^, POLATOM, Otwock, Poland) in accordance with the manufacturer’s instructions. Briefly, freshly eluted ^99m^TcO_4_^-^ (740 MBq) in a 0.9% NaCl solution (pH 7) was added to vials containing HYNIC-Tyr3-octreotide (20 µg), tricine, and EDDA, mixed and incubated at 80 °C for 30 min according to existing recommendations [30].

### 2.4. Imaging Procedures

#### ^99m^TcHYNIC-TOC Scintigraphy

Patients were intravenously injected with ^99m^TcHYNIC-TOC. The injected dose was standard 370 MBq. Planar and SPECT images were acquired using a dual-head gamma camera (Infinia, GE Healthcare, Milwakee, WI, USA) equipped with double-head, high-resolution, low-energy parallel hole collimators. Planar images of the head, neck, and joints were acquired for 10 min starting 3 h after intravenous injection of the radiotracer in a 512 × 512 matrix. The SPECT study was performed using 120 projection images over a 360° rotation, with 20 s per projection in a 128 × 128 matrix. Transaxial, coronal, and sagittal tomograms were reconstructed using a Butterworth filter and the ordered subset expectation maximization (OSEM) algorithm (two iterations, 10 subsets). ×100.

### 2.5. Image Analysis

The ^99m^TcHYNIC-TOC images were analysed by two observers (KA and SR) who were independent of each other and blinded to the clinical details. Analysis was performed using a previously proposed method (22). We did not consider the hips because of possible artefacts due to high bladder activity. The 99mTcHYNIC-TOC images were analysed qualitatively via calf uptake as a reference background. Uptake greater than calf uptake was considered abnormal, according to previous reports. To compare the data from the 7 patients who underwent explantation, we used the semiquantitative analysis reported by our group in previous studies (0, 1, 2, 3, and 4 compared with calf) [30].

### 2.6. Statistical Analysis

Patient demographics and clinical characteristics, including frequencies, percentages, means, and medians, were summarized using descriptive statistics. The associations between clinical symptoms and scintigraphic findings were evaluated using Matthews correlation coefficients. The pre- and postexplantation scintigraphic results were compared via paired analysis of seven patients who underwent repeat imaging following explantation. All the statistical analyses were performed using Stata 14.2 SE.

## 3. Results

Data were collected from 50 participants with SBIs who exhibited ASIA symptoms, with a mean age of 45.5 years (range: 24–67 years). Seven patients underwent explantation, followed by ^99m^TcHYNIC-TOC scintigraphy at a median of 18 months postsurgery. The demographic characteristics of the participants are summarized in Table 1.

**Figure 1:**
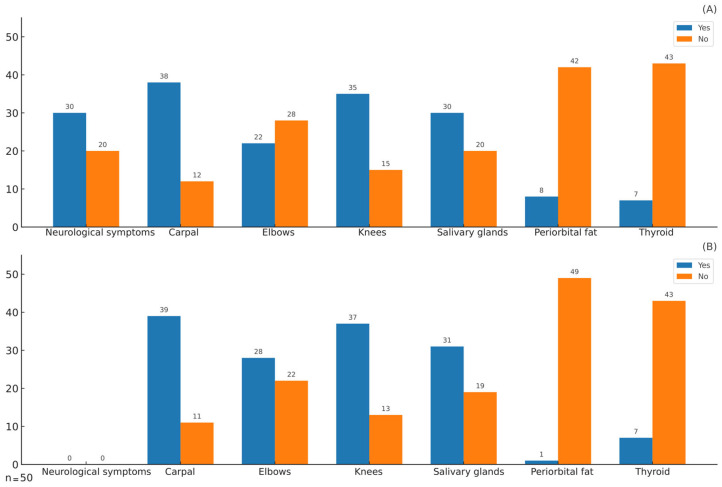
Frequency of symptoms and findings on ^99m^TcHYNIC-TOC scintigraphy among patients with SBIs and autoimmune symptoms. (**A**) Symptoms. (**B**) Positive findings on ^99m^TcHYNIC-TOC scintigraphy.

The frequency of symptoms, which included thyroid dysfunction, periorbital fat changes, sicca, carpal and knee pain, and neurological symptoms, along with the corresponding findings from ^99m^TcHYNIC-TOC scintigraphy, are illustrated in Figure 1. The most frequently reported symptoms were associated with carpal and knee pain and sicca syndrome, which were correlated with the most frequent and highest abnormal uptake observed via HYNIC-TOC scintigraphy.

The correlations between reported symptoms and scintigraphic findings are presented in Table 2, highlighting the strongest associations between knee pain and sicca symptoms with positive scintigraphic results in the corresponding regions. These relationships were quantified by Matthews correlation coefficients of 0.81 and 0.92, respectively.

**Figure 2:**
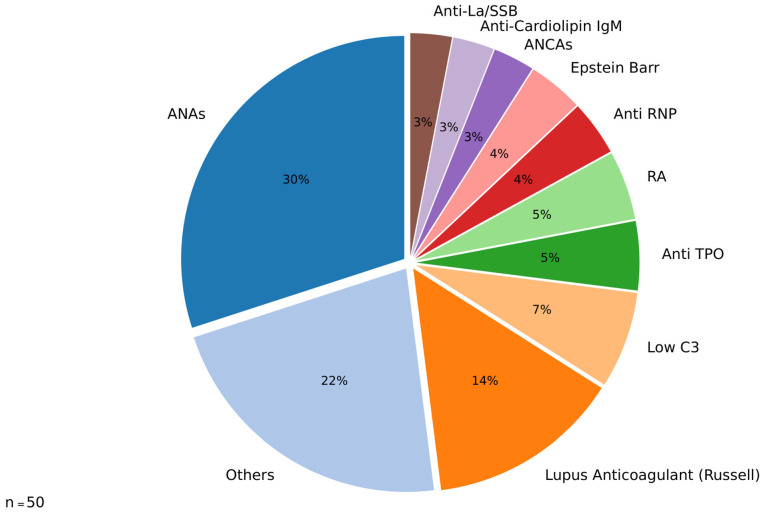
Antibody distribution in patients with silicone implant-associated autoimmunity.

The distribution of specific antibodies detected in 31 out of 50 patients with SBIs and autoimmune-like symptoms is illustrated in Figure 2. The data highlight a predominance of ANAs among the identified antibodies, accounting for 30% of positive cases, followed by lupus anticoagulant (Russell, 14%); the group categorized as “others” includes antibodies that were detected at very low frequencies and, for data management purposes in the pie chart, were consolidated into a single category.

**Figure 3:**
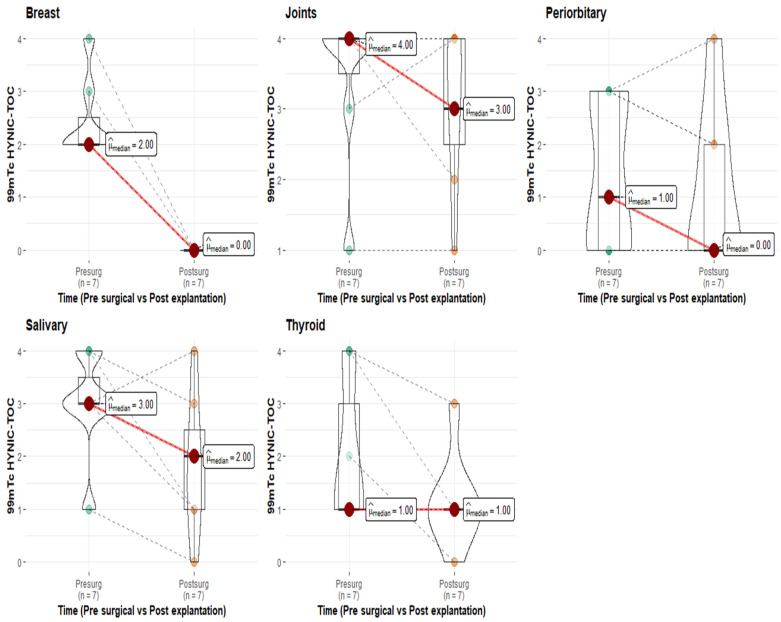
Semiquantitative analysis of pre -and postsurgical ^99m^Tc HYNIC-TOC scintigraphy.

A semiquantitative analysis of findings in seven patients before and after explantation is provided in Figure 3, while details of the frequency of improvements observed in ^99m^TcHYNIC-TOC scintigraphy postsurgery are provided in Table 3. 

**Figure 4:**
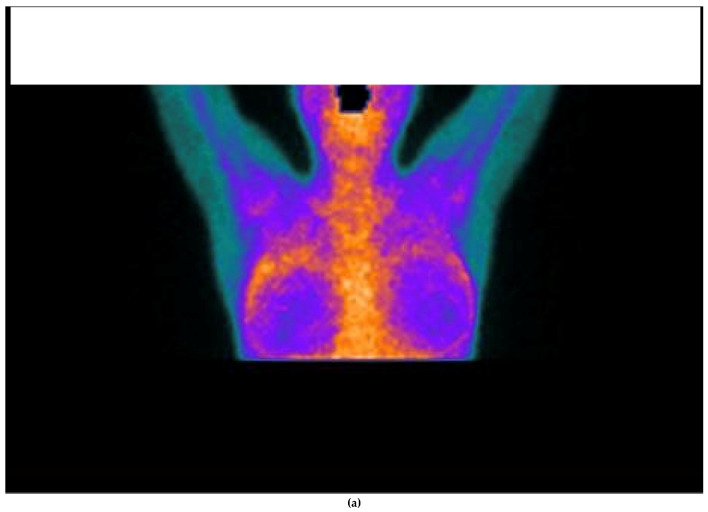

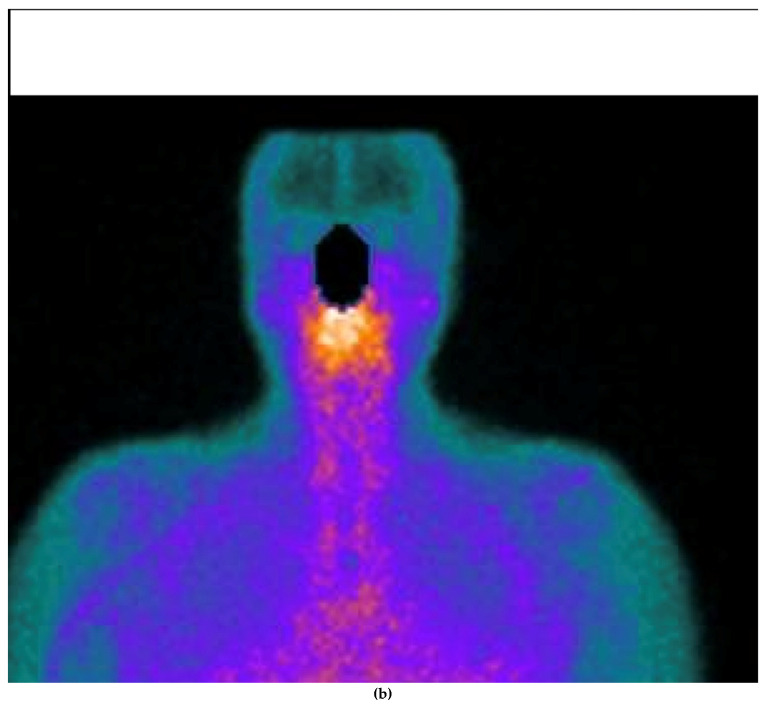

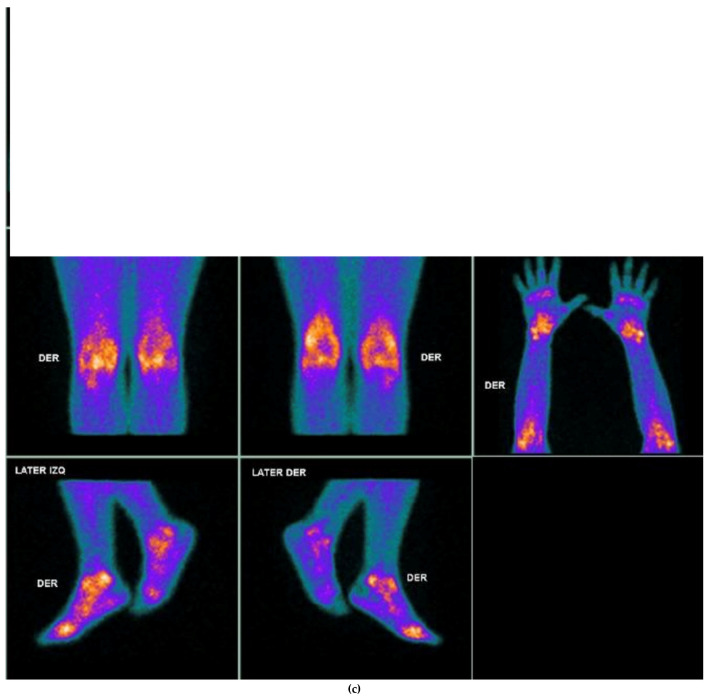
^99m^Tc HYNIC-TOC scintigraphy showing abnormal uptake of the radiotracer surrounding the breast implants (**a**) and knees, elbows, carpal joints, and ankles (**c**). Postexplantation scintigraphy showing no uptake in surgical bed in the same patient (**b**).

Notably, all explanted patients exhibited no abnormal uptake of ^99m^TcHYNIC-TOC in the surgical bed. However, the postoperative patterns in the other evaluated organs varied. Thyroid uptake remained unchanged in five of the seven patients, whereas joint findings improved in three patients and remained stable in another three patients. Similarly, salivary gland findings improved in three patients, with four remaining stable. Notably, all but one patient tested positive for autoantibodies associated with autoimmune conditions (Figure 4).

## 4. Discussion

This study evaluated, for the first time, the characteristic uptake patterns of ^99m^TcHYNIC-TOC scintigraphy in patients with SBIs and autoimmune-like symptoms. The study confirmed significant scintigraphic uptake in organs associated with reported autoimmune symptoms, particularly in the joints and salivary glands. The findings revealed strong correlations between clinical symptoms, such as knee pain and sicca syndrome, and abnormal scintigraphic uptake in the corresponding regions, with Matthews correlation coefficients of 0.81 and 0.96, respectively. Additionally, the comparative analysis of pre- and postexplantation imaging demonstrated that explantation effectively resolved abnormal uptake in the surgical bed in all patients, with high variability in uptake changes across other organs, such as the thyroid, joints, and salivary glands, which underscores the heterogeneity of the inflammatory response associated with ASIA syndrome.

A considerable proportion of patients with silicone-related disease fulfil the diagnostic criteria for fibromyalgia [31], undifferentiated connective tissue disease [32], and sarcoid-like disease [33]. Additionally, many of these patients exhibit well-defined systemic autoimmune diseases, including Sjögren’s syndrome, rheumatoid arthritis, systemic sclerosis, antiphospholipid syndrome, and various forms of vasculitis [34]. These underlying autoimmune processes explain commonly reported symptoms, such as chronic fatigue, early-onset arthritis (observed in more than 90% of cases), myalgias (affecting up to 90% of patients), sicca symptoms (present in the majority of cases) [15], and severe neurological manifestations, which occur in 30–40% of patients. In the present cohort, the most frequent symptoms were articular, neurological, and sicca manifestations, as detailed in Table 1 and illustrated in Figure 1A. Notably, the regions of abnormal uptake detected by SSTR imaging using ^99mTc^HYNIC-TOC coincided with symptomatic sites, particularly the joints and salivary glands, as shown in Figure 1B. Furthermore, Matthews correlation analysis confirmed a strong association between abnormal imaging findings and patient-reported symptoms (Table 2). The overlap between symptoms and abnormal imaging findings can be attributed to the role of SST and SSTRs in autoimmune-driven inflammation. During autoimmune inflammation, activated lymphocytes and macrophages exhibit increased expression of SSTRs, which are also found in neoangiogenic vessels, proliferating synovial vessels, and epithelioid cells [35]. This receptor overexpression reflects localized inflammatory activity, particularly in the joints and salivary glands, which is consistent with the symptomatic regions reported by patients. In a few patients, a discrepancy was observed between periorbital symptoms and radiotracer uptake, which may be explained by the variable expression or activation of somatostatin receptors on periorbital fibroblasts. Radiolabelled SST analogues have demonstrated utility in single-photon emission computed tomography (SPECT) and positron emission tomography (PET) for chronic inflammatory diseases, providing clinically relevant insights into disease activity, prognosis, and therapeutic response [26,27]. Molecular imaging’s capability to visualize active inflammation via targeted SSTR expression provides a clear biological basis for symptoms of autoimmune conditions and highlights its diagnostic value in identifying and monitoring complex inflammatory disorders.

Autoantibodies are a defining feature of autoimmune diseases. Experimental evidence has demonstrated their role in silicone-related conditions. For example, a study in mice that spontaneously developed autoimmune diseases following silicone implantation revealed a significant increase in anti-dsDNA antibodies (*p* < 0.02), rheumatoid factor, and silicone-bound autoantibodies [36]. The ability of silicone to promote autoimmunity may stem from its hydrophobic surface, which alters antigen conformation, leading to immune system recognition of these modified structures and triggering an autoimmune response [37,38]. Additionally, cross-reactivity between silicone and glycosaminoglycans in connective tissue has been proposed as another mechanism of immune activation [13]. Clinical studies further support this link. In a cohort of 57 patients in California, 35% of women with SBIs tested positive for anti-collagen autoantibodies, a prevalence comparable to that observed in patients with erosive arthritis [13]. Similarly, Zandman-Goddard et al. reported elevated levels of 15 distinct autoantibodies in patients with SBIs, regardless of their symptomatology (122 symptomatic vs. 86 asymptomatic women). Among these, anti-SSB/La and anti-collagen II antibodies were the most predominant and were significantly elevated in both groups [39]. Moreover, a large cohort study involving nearly 400,000 women, including 11,800 with SBIs, identified an increased relative risk of 1.24 (95% CI, 1.08–1.41; *p* = 0.0015) for developing connective tissue disorders [40]. Serologic assessments in women with SBIs have also revealed isolated reductions in complement components C3 and C4 [41]. In the present study, a clear pattern of autoimmune activation was observed, with 33 out of 50 patients (66%) testing positive for at least one autoantibody (Figure 2). Among these, antinuclear antibodies (ANAs) were the most frequently detected (30%), followed by lupus anticoagulant Russell (14%), decreased complement component C3 (7%), and anti-thyroid peroxidase (Anti-TPO) antibodies (5%), among others. These findings are consistent with the broader literature linking silicone exposure to the development of autoantibodies and systemic immune dysregulation, as described in the autoimmune/inflammatory syndrome induced by adjuvants (ASIA). Silicone is thought to act as an immunological adjuvant that facilitates the breakdown of self-tolerance, leading to humoral and cellular immune activation [1]. Importantly, these serologic findings were paralleled by in vivo molecular imaging data: most patients with symptoms demonstrated increased uptake on 99mTc-HYNIC-TOC scintigraphy, particularly in the joints and salivary glands—regions commonly involved in autoimmune processes. This uptake reflects the overexpression of somatostatin receptors (SSTRs) on activated lymphocytes and macrophages, which are known to accumulate in autoimmune inflammation. While SSTR overexpression can occur in other inflammatory conditions, including infectious, neoplastic, and granulomatous diseases [22], the concordance in our cohort between symptom location, autoantibody positivity, and SSTR uptake supports the hypothesis that the immune activation in these patients is autoimmune in nature. Together, these findings underscore the potential of SSTR-targeted scintigraphy as a noninvasive tool to identify organ-specific autoimmune inflammation in patients with SBIs and suspected ASIA, complementing serologic and clinical data in diagnostic evaluation. Seven patients in the cohort underwent surgery, and their baseline and postexplantation data were compared. A key finding was the resolution of abnormal ^99m^TcHYNIC-TOC uptake in the surgical bed postsurgery. In contrast, findings in other organs—such as the thyroid, joints, and salivary glands—showed no consistent changes among patients, suggesting variable systemic inflammatory responses. These results highlight the role of explantation in reducing localized inflammation, as evidenced by the absence of abnormal uptake in the surgical bed. Given the limit in this subgroup, these findings should be interpreted as preliminary and exploratory; larger prospective studies are needed to validate these postexplantation observations. Previous reports have emphasized that explantation is an important first step in the management of patients with ASIA due to an implant; unfortunately, several women still suffer from ASIA after explantation [42,43]. This variability underscores the complexity of the syndrome and the need for a more comprehensive management strategy. Clinically, the observed findings support the potential use of ^99m^TcHYNIC-TOC scintigraphy as a valuable tool for assessing organ-specific inflammation. While explantation effectively addresses localized inflammation, the ongoing symptoms in some patients highlight the heterogeneity of ASIA syndrome and its systemic impact. Further research involving larger cohorts and prospective studies is needed to validate these observations and better understand the mechanisms driving organ-specific inflammatory responses postexplantation.

The evidence linking SBIs to ASIA is steadily accumulating. Recognizing this association underscores the importance of identifying women who may have an increased risk of developing this condition [8]. A genetic predisposition to autoimmunity is a plausible factor, with silicone potentially acting as an environmental trigger. However, as such predispositions are not yet easily diagnosable, it is prudent to consider potential risks in defined at-risk groups before deciding on the use of SBIs. Identifying these risk groups can help minimize health complications associated with autoimmune disorders. Furthermore, the associations between SBIs and inflammatory markers highlight the need for robust preoperative and postoperative protocols to assess potential autoimmune risks. Although inflammatory markers alone do not confirm specific immunological diseases, strategic testing combined with interdisciplinary consultations can aid in effective risk management. Collaboration among healthcare providers from different specialties is crucial for addressing the multifaceted risks associated with SBIs. The role of molecular nuclear medicine in this context is particularly significant, as functional imaging can detect in vivo components and phases of diseases, including inflammation-related conditions. This capability extends beyond the morphological abnormalities typically identified by conventional radiological methods, providing a strong rationale for employing nuclear medicine techniques in early diagnosis and treatment planning.

This study provides valuable insights into the role of SSTR scintigraphy as a tool for assessing SST-mediated inflammation and the impact of explantation on inflammatory activity in patients with ASIA syndrome. While previous reports have focused primarily on systemic inflammatory responses in patients with SBIs, our study adds to the literature by offering semiquantitative evidence through SSTR imaging ^99m^TcHINIC-TOC.

The key strengths of this study include the use of Matthews correlation coefficients to quantify associations between clinical symptoms and scintigraphic findings, as well as paired pre- and postexplantation analyses, which allow for a more nuanced understanding of changes in inflammatory activity. However, several limitations must be acknowledged. The relatively small sample size of 50 patients, coupled with the small postexplantation cohort and retrospective design, restricts the generalizability of the findings and may introduce bias. It is important to note that although all patients presented with clinical symptoms and/or serological markers suggestive of immune dysregulation at the time of scintigraphy, comprehensive data regarding pre-existing autoimmune conditions prior to breast implantation were not consistently available. In a subset of cases, personal or familial autoimmune predispositions were reported, in line with the known risk profiles for ASIA syndrome. However, due to the retrospective nature of the cohort and the referral-based sample, this information was incomplete and represents a limitation. Future prospective studies should aim to systematically document pre-implantation immune status to better delineate causality and risk stratification.

Future prospective studies involving larger cohorts are necessary to validate these results and to further explore the long-term clinical outcomes of explantation in patients with ASIA syndrome. Such investigations could provide a more comprehensive understanding of the relationship between SBIs and SST-mediated inflammation, ultimately enhancing clinical decision making and patient care.

## 5. Conclusions

This study highlights the potential of ^99m^TcHYNIC-TOC SSTR scintigraphy as a valuable tool for assessing SST-mediated inflammation and the impact of explantation in patients with ASIA syndrome. This research underscores the importance of multidisciplinary approaches, robust preoperative risk assessments, and innovative imaging techniques in optimizing the management of ASIA syndrome and related autoimmune conditions. Future prospective studies with larger cohorts are warranted to validate these results, elucidate the mechanisms underlying silicone-induced autoimmunity, and explore long-term outcomes of explantation.

## Figures and Tables

**Table 1:** Demographic characteristics (*n* = 50).

Characteristics ^a^	Total, *n* (%) *n* = 50
Age in years	44.50 (37.00–51.00)
Age group	
20–30	1 (2.00%)
30–40	14 (28.00%)
40–50	19 (38.00%)
50–60	12 (24.00%)
≥ 60	4 (8.00%)
Consultation symptoms	
Neurological symptoms	30 (60.00%)
Carpal	38 (76.00%)
Elbows	22 (44.00%)
Knees	35 (70.00%)
Sicca	30 (60.00%)
Periorbital fat	8 (16.00%)
Thyroid gland	1 (2.00%)
Scintigraphic findings	
Carpal	39 (78.00%)
Elbows	28 (56.00%)
Knees	37 (74.00%)
Salivary glands	30 (60.00%)
Thyroid gland	12 (24.00%)

Notes: ^a^ Data are presented as the mean (SD) for continuous measures, and *n* (%) for categorical measures.

**Table 2:** Matthews correlation coefficients between reported symptoms and scintigraphic findings.

Characteristics ^a^	Gammagraphic Findings Negative	Gammagraphic Findings Positive	Total	*ϕ* Matthews’s Correlation	*p* Value
	***n* = 11**	***n* = 39**	***n* = 50**		
Carpal symptoms				0.72	<0.001
Negative	9 (82%)	3 (8%)	12 (24%)		
Positive	2 (18%)	36 (92%)	38 (76%)		
	***n* = 22**	***n* = 28**	***n* = 50**		
Elbow symptoms				0.79	<0.001
Negative	22 (100%)	6 (21%)	28 (56%)		
Positive	0 (0%)	22 (79%)	22 (44%)		
	***n* = 13**	***n* = 37**	***n* = 50**		
Knee symptoms				0.81	<0.001
Negative	12 (92%)	3 (8%)	15 (30%)		
Positive	1 (8%)	34 (92%)	35 (70%)		
	***n* = 49**	***n* = 1**	***n* = 50**		
Periorbitary symptoms				0.33	0.37
Negative	42 (86%)	0 (0%)	42 (84%)		
Positive	7 (14%)	1 (100%)	8 (16%)		
	***n* = 19**	***n* = 31**	***n* = 50**		
Salivary glands symptoms				0.92	<0.001
Negative	18 (95%)	1 (3%)	19 (38%)		
Positive	1 (5%)	30 (97%)	31 (62%)		

^a^ Data are presented as *n* (%) for categorical measures.

**Table 3:** Frequency of organ improvement under ^99m^TcHYNIC-TOC in patients who underwent explantation.

Characteristics ^a^	Improvment
	*n* = 7
Implants	7 (100%)
Thyroid	1 (14.28%)
Joints	3 (42.85%)
Salivary glands	3 (42.85%)
Periorbitary fat	2 (28.57%)

^a^ Data are presented as *n* (%) for categorical measures_._

## Data Availability

The original contributions presented in this study are included in the article. Further inquiries can be directed to the corresponding author.
